# Dual mechanisms give rise to biphasic stomatal opening in onion

**DOI:** 10.1111/nph.71615

**Published:** 2026-10-06

**Authors:** Melissa Tomkins, Matthew J. Wilson, Jodie V. Armand, Nathanael Y. H. Tan, Briony Parker, Julie E. Gray, Andrew J. Fleming, Richard J. Morris, Richard S. Smith

**Affiliations:** ^1^ Computational and Systems Biology John Innes Centre Norwich Research Park Norwich NR4 7UH UK; ^2^ Plants, Photosynthesis and Soils, School of Biosciences University of Sheffield Western Bank Sheffield S10 2TN UK

**Keywords:** biomechanics, FEM modelling, guard cells, imaging, onion, stomata

## Abstract

- Stomata are controllable micropores on the leaf surface that regulate gas exchange and water loss. Pore aperture adjustment is a mechanical process whereby changes in guard cell turgor drive changes in cell shape, resulting in stomatal opening or closure.
- Previous computational models of kidney‐shaped stomata primarily relied on guard cells elongating upon turgor increase, a process dependent on cell wall properties, such as anisotropy in stiffness, strain‐stiffening, and polar fixing.
- Finite element method simulations using experimentally derived 3D meshes from confocal imaging of *Allium cepa* (onion) suggest an alternative mechanism for opening that is largely due to changes in guard cell cross‐sectional shape. This mechanism does not rely on the assumptions for cell wall parameters required in previous models based on idealised geometries.
- We propose that mechanical responsiveness of guard cells to turgor changes is determined by a combination of two processes: one dependent on shape and the other on wall material properties. This suggests the parameters governing guard cell dynamics can be viewed abstractly as a two‐dimensional (shape and material properties) ‘morphospace’, allowing multiple routes of shape adaptation to optimise gas exchange.

## Graphical abstract

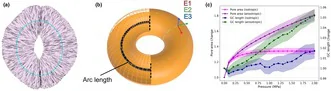

## Introduction

Stomata are microscopic pores on the plant epidermis that play a critical role in regulating gas exchange in and out of the plant (Hetherington & Woodward, 2003; Lawson & Matthews, 2020). Changes in stomatal aperture influence both CO_2_ uptake for photosynthesis and water loss through transpiration. Each stomate is defined by a pair of guard cells whose turgor pressure dynamically changes with osmolyte concentration, governed by ion fluxes, such as potassium (K^+^) and chloride (Cl^−^), that mediate pore opening and closure. These ion fluxes are induced by environmental cues, such as light intensity, CO_2_ concentration, water availability, and pathogen attacks, as well as internal signals, including the stress hormone abscisic acid. Solute accumulation within guard cells increases turgor pressure through osmosis, leading to stomatal opening, thereby enhancing photosynthesis and transpiration. Several stress conditions trigger solute efflux, reducing turgor pressure and promoting stomatal closure, thereby limiting water loss and pathogen accessibility but also photosynthesis. Although the biochemical and ion transport systems are largely conserved across land plants (Clark *et al*., 2022), there has been specialisation in guard cell form in the angiosperms. The majority of land plant species produce kidney‐shaped guard cells, but the grasses have dumbbell‐shaped guard cells, which are always accompanied by lateral subsidiary cells.

The inflation and deflation of guard cells, along with the corresponding change in stomatal pore aperture, are fundamentally mechanical processes (Franks & Farquhar, 2007; Woolfenden *et al*., 2018). Early observations of guard cell geometry and cell wall properties, particularly wall thickness (Von Mohl, 1856), were later extended into the first mechanical interpretations for stomatal opening by Schwendener (1881) and Haberlandt (1884) who described differential wall thickness and curvature changes. The later interpretation of directional mechanical constraint arising from oriented cellulose microfibrils was developed in the mid‐20th century (e.g. Ziegenspeck, 1938) and subsequently incorporated into mechanistic classifications of stomatal function (e.g. Guttenberg, 1959). Consequently, substantial research has focused on identifying the cell wall properties and guard cell geometries that influence stomatal efficiency – here defined as the increase in pore area for a given increase in pressure. The direction of expansion in plant cell walls depends upon the orientation of stiff cellulose microfibrils, which confer direction‐dependent (anisotropic) stiffness. In guard cells of ferns and angiosperms, these microfibrils follow curved trajectories within the cell wall, such that their local orientation is predominantly radial with respect to the stomatal pore. By contrast, in grass stomata, microfibrils in the narrow central rod region are oriented parallel to the long axis. However, observations differ for the bulbous ends: early reports described a radial arrangement (Shtein *et al*., 2017) whereas more recent work suggests a predominantly longitudinal orientation (Wilson *et al*., 2025). Previous research has explored the relative importance of anisotropic stiffness of guard cell walls, along with turgor pressure, guard cell wall thickness, mechanical influence of neighbouring cells, outer stomatal ledges (Pautov *et al*., 2017, 2019) and guard cell geometry (reviewed in Woolfenden *et al*. (2018) and Tan *et al*. (2025)). Early work on stomatal biomechanics focused on the concept of ‘mechanical advantage’, in which increases in guard cell turgor pressure cause them to push outwards against the surrounding epidermal cells. The mechanical resistance of the epidermis was proposed to make the guard cells bend, rather than expanding uniformly, increasing the stomatal pore size (DeMichele & Sharpe, 1973). The bending effect was proposed to be enhanced by the presence of a thicker ventral (inner) guard cell wall (Meidner & Mansfield, 1968), in line with the historical observations (Schwendener, 1881; Haberlandt, 1884; Guttenberg, 1959).

However, Aylor *et al*. (1973) challenged the idea that asymmetric guard cell wall thickness is essential for stomatal opening. Using inflated balloons and masking tape to mimic both guard cell geometry and microfibril stiffening, and dorsal‐ventral wall thickness differences, they showed that a difference in thickness between the ventral and dorsal walls is not strictly required for stomatal opening. Instead, they proposed that anisotropic stiffness of the guard cell wall and a fixed stomate length were of greater importance. Cooke *et al*. (1976) introduced a more realistic finite element method (FEM) shell model that included the effect of surrounding epidermal cells and allowed changes in guard cell cross‐sectional shape. Their simulations agreed with Aylor *et al*. (1973) in that a thicker ventral wall was not necessary for stomatal opening. However, Cooke *et al*. (1976) found that anisotropic stiffness increased stomatal opening by amplifying pressure differences between guard cells and neighbouring cells, producing a cross‐section that bulged out of the leaf plane, changing from a wide, low oval when deflated to a tall, narrow oval shape when inflated. Sharpe & Wu (1978) further emphasised the importance of guard cell cross‐sectional geometry, particularly regarding the way this geometry changes towards the poles of the stomatal complex.

Despite these early advances, many modelling studies have relied on the use of idealised, toroid representations of stomata, reflecting a paucity of information concerning the 3D shape of the guard cells. For example, using hollow, deformed toric meshes of guard cells with circular cross‐sections, we previously showed through FEM modelling that both cell wall anisotropy and strain‐stiffening are essential for capturing observed pressure–aperture relationships in stomata with kidney‐shaped guard cells (Woolfenden *et al*., 2017). While microfibrils are locally radial with respect to the stomatal pore, they curve around the guard cells, stiffening the wall against circumferential expansion, creating circumferential anisotropic stiffness. Hereafter we use ‘circumferential stiffness’ to refer to this direction‐dependent mechanical resistance. Without circumferential stiffness of the guard cell walls, these models were unable to open the pore and, indeed, the pore closed upon inflation. Under these conditions, the main mechanism for the opening of the stomatal pore was cell elongation (arc length) driven by cell wall anisotropy (Meckel *et al*., 2007; Woolfenden *et al*., 2017). Carter *et al*. (2017) proposed that, as previously suggested by Von Mohl (1856) and later by Aylor *et al*. (1973), if the length of the stomatal complex was constrained, this could enhance opening by forcing the cells to deflect more towards the sides in response to increasing arc length.

In parallel to progress on computational modelling, experimental approaches have advanced our understanding of stomatal biomechanics, generally with a focus on how guard cell wall composition influences mechanical behaviour. For example, Rui *et al*. (2016) investigated the effect of cellulose and xyloglucan levels on stomatal function, Shtein *et al*. (2017) compared cell wall composition and structural patterns between different plant species, and Keynia *et al*. (2023) compared stomatal opening efficiency between wild‐type Arabidopsis and mutants with differences in cellulose content and pectin molecular weight. While these studies have highlighted the importance of guard cell wall structure, the role of guard cell geometry has been less well investigated.

Recently, we reported on stomatal mechanics using realistic 3D geometries of dumbbell‐shaped guard cells found in specialised grass stomata. By combining confocal microscopy with FEM modelling, we demonstrated that geometry plays an important role in determining grass stomata opening efficiency, with less reliance on specialised or complex wall material properties (Durney *et al*., 2023). These findings have prompted us to return to kidney‐shaped guard cells and to reassess their behaviour using more realistic geometries than those implemented in previous studies. In particular, advances in imaging and mesh reconstruction now allow the creation of 3D meshes directly from confocal microscopy data, thus allowing a closer simulation of actual guard cell shape.

Here, we compare FEM models based on geometries extracted from 3D confocal microscopy (hereafter: experimental meshes) of onion stomata with FEM models using geometries generated parametrically (hereafter: idealised meshes). Despite having kidney‐shaped guard cells, stomata in onion are arranged in files along the leaf, as in grasses. We reasoned that comparing different guard cell shapes within a similar stomatal arrangement would give a more direct comparison of the role of guard cell geometry between species, without the addition of different spatial patterning. We show that when experimentally derived kidney‐shaped guard cell geometries are used, the resultant FEM simulations show that guard cell pore opening can occur without the requirement for anisotropic cell wall properties and/or strain‐stiffening, both of which were previously shown to aid opening in Arabidopsis (Woolfenden *et al*., 2017). These findings led us to reassess the importance of geometry in stomatal function. We find that, similar to the biphasic opening previously observed by Stålfelt (1927) and later further developed by Sharpe & Wu (1978), onion stomata open through the combination of two mechanisms: a change in cross‐sectional shape, moving the guard cells from oval to circular; subsequent lengthening and bending due to anisotropic cell wall stiffness. We propose that there is a flexibility in the combination of geometry and cell wall material properties with respect to stomatal function, findings of potential importance for understanding stomatal evolution.

## Materials and Methods

### Experimental mesh extraction

#### Plant material and growth conditions

Seeds of onion (*Allium cepa* L., cv White Lisbon) were sown into Levington M3 compost and placed into a controlled environment growth chamber (16 h : 8 h, 21°C : 16°C, light : dark, 400 μmol m^−2^ s^−1^ photosynthetic photon flux density, 60% relative humidity). Seven to nine days after sowing, onion seedlings were transplanted to 1 l pots filled with a 3 : 1 (v/v) ratio of M3 and perlite, plus 5 g of solid slow‐release fertiliser. Plants were kept well watered until leaf tissue was harvested.

#### Confocal imaging and segmentation

Onion leaf samples were prepared for confocal microscopy as described in Wilson *et al*. (2025). Onion leaf samples *c*. 10 mm in length were excised from the fully expanded fifth leaf and immediately plunged into 3 : 1 ethanol : acetic anhydride (v/v) before vacuum infiltration for 1 h. Samples were kept in fixative at 4°C for 48 h, rinsed in 50% ethanol, and transferred to 70% ethanol for storage. When required, samples were removed from ethanol and subjected to a short treatment in chloroform to remove leaf waxes. Samples were next progressively rehydrated via an ethanol series before they were bleached and treated with amylase. Cell walls were stained with pseudo‐Schiff propidium iodide (PI) and samples were cleared in a solution of chloral hydrate and glycerol. Finally, samples were cut and mounted on slides in Hoyer's solution.

Images of onion stomata were acquired using a Zeiss LSM880 Airyscan confocal microscope in LSM mode using a 20× air objective (Plan‐Apochromat 20×, NA: 0.8). PI‐labelled cell walls were visualised using a 561 nm laser and a 488/561 dichroic mirror. Image resolution was set to 488 × 488 pixels and scan speed was set at the maximum. Pinhole size was kept constant at 1 AU. Z‐step size was fixed at 0.3 μm and each stack was set up to include several microns above and below each stomate. After collection, images were subjected to AiryScan Processing (Zen; Zeiss). Eighteen stomata were imaged across three biological replicates.

Meshes of onion guard cell geometry were generated as previously described (Wilson *et al*., 2025). Cell segmentation was performed using MorphographX (MGX) (Strauss *et al*., 2022). Stacks were imported into MGX and initially brightened using the ‘Stack/Filter/Brighten Darken’ process. Cell boundaries were then predicted using the process ‘Stack/CNN/UNet3D’. Following boundary detection, a Gaussian blur filter with a radius of 0.3 μm was applied to the stack. Segmentation of images was carried out using the ‘Stack/ITK/Segmentation/ITK Watershed Auto Seeded’ function with a threshold of 500. As the watershed algorithm identifies the midpoint of the cell wall signal, the segmentation line is shifted slightly from the extremity of the true boundary. Over‐segmentation errors were corrected and any excess labels (i.e. epidermal pavement cells, mesophyll cells, and intercellular air spaces) not corresponding to the guard cells were manually deleted from each stack. Meshes of cell geometry were created using ‘Mesh/Creation/Marching Cubes 3D’ process with a cube size of 1 μm and three smooth passes. Where required to better represent cell shape, meshes were smoothed further using the process ‘Mesh/Structure/Smooth Mesh’. Meshes were rescaled in the Z‐direction as described previously (Diel *et al*., 2020) to account for refractive index differences associated with image acquisition. Mesh files were used directly to inform finite element analysis in MorphomechanX (MMX, https://morphomechanx.org/). The onion stomata were closed at the time of imaging, so any apparent gap between guard cells reflects the way that the segmentation algorithm works in identifying the mid‐point of the guard cell wall, rather than indicating an open pore.

### Computational modelling

We focused on modelling the dynamics of isolated guard cells, neglecting the impact of surrounding cells. In all cases, stomata were modelled as hollow shells partitioned into two guard‐cell compartments, with a wall thickness of 1 μm. Simulations were performed in the MMX framework, following the methods previously described in Durney *et al*. (2023) and Wilson *et al*. (2025). FEM was used to find the mechanical equilibrium after applying turgor pressure to the walls of the stomatal complex. The resulting deformation of the starting mesh depends on parameters defining the material properties of the elastic cell wall. The cell walls were modelled as a transversely isotropic Saint‐Venant Kirchhoff material with two Young's moduli (E1 = E3, E2). The Poisson's ratio was captured by a single parameter approximating the compressibility. For the simulations with anisotropic stiffness, the Young's modulus E2 defines the material stiffness in the direction of the cellulose microfibril primary orientation (Fig. 4a,b). The other Young's modulus, E1 and E3, defines the material stiffness in the orthogonal directions. For the isotropic simulations E1 = E3 = E2.

The increase in internal turgor pressure (2 MPa) was chosen based on the point at which we observed a measurable overall size increase in our meshes and falls within previously published estimates of guard cell turgor (0.5 to 5 MPa) (Franks *et al*., 2001). Turgor pressure is applied as a load normal to the triangular elements of the mesh. The model provides the deformation of the stomatal mesh and the stress and strain distributions within the complex. These outputs reflect the combined effects of geometry, material properties, and turgor pressure on stomatal mechanics. From the resulting geometry, we calculate the pore area, along with the guard cell arc length and changes to the cross‐section aspect ratio, defined as the ratio of the length of the major axis (longest dimension) to that of the minor axis (orthogonal shortest dimension).

Mesh selection was based on the pore length‐to‐stomatal length ratio. Meshes with pores spanning < 25% of the stomatal length were excluded to avoid cases where the segmentation did not clearly separate the two guard cells at the pore, resulting in artificially connected geometries. All mesh measurements were extracted automatically, in order to maintain consistency and reproducibility (see Supporting Information Methods S1). Model parameters are given in Table 1.

**Table 1:** Material property parameters used in the onion guard cell simulations.

Parameter	Value	Source
Poisson's ratio	0.3	Wilson *et al*. (2025)
Young's modulus	E1/E3 100 MPa E2 100 MPa (isotropic) 1000 MPa (anisotropic)	Carter *et al*. (2017); Woolfenden *et al*. (2017); Wilson *et al*. (2025)
Pressure increase	2 MPa	This study; Franks *et al*. (2001)

E1/E3 and E2 are the two Young's moduli of the transverse isotropic cell wall; E2 is in the circumferential direction (the primary cellulose microfibril orientation) and E1/E3 in the orthogonal directions.

## Results

### FEM models of onion stomata using confocal segmented geometry open without anisotropic stiffness or polar pinning

To test the behaviour of FEM simulations using realistic guard cell geometries, we extracted meshes from confocal light microscopy images of closed onion stomata, following Durney *et al*. (2023) and Wilson *et al*. (2025) (see the Materials and Methods section) (Fig. 1a,b). To investigate how these experimentally derived 3D shapes respond to turgor pressure, we constructed an FEM model in MMX and simulated its deformation under an internal turgor pressure increase from 0 to 2 MPa. We initially used an isotropic Saint‐Venant material model with a Young's modulus of 100 MPa, a Poisson's ratio of 0.3, and an element thickness of 1 μm. The cell wall parameters and final turgor pressure were selected so that the model reproduced the observed deformation. The increase in turgor pressure caused the guard cells to expand, altering their morphology and opening the stomatal pore. Material parameters were not changed between simulations; however, the qualitative results were insensitive to their specific values (see Fig. 1e).

**Fig. 1:**
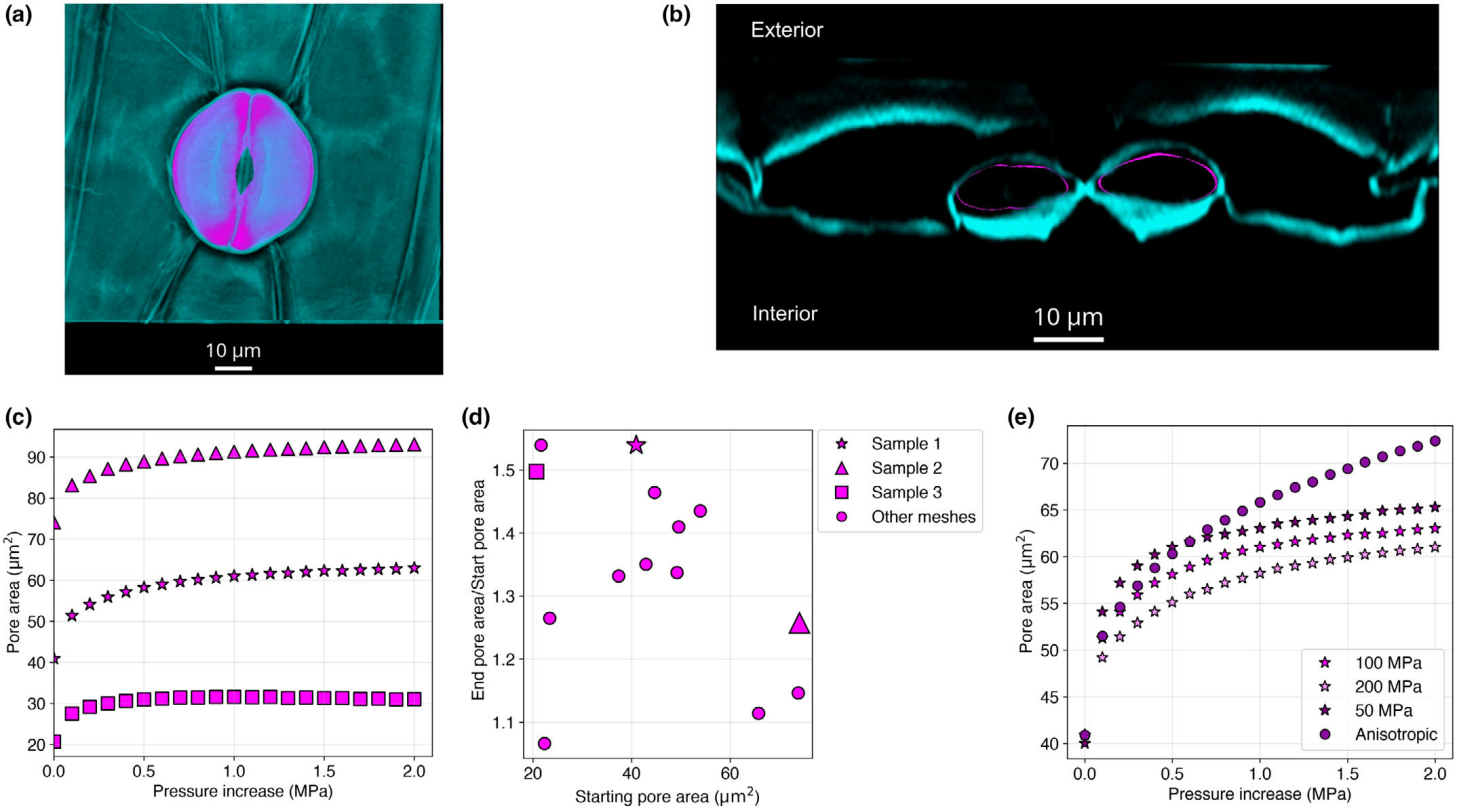
Finite element method (FEM) models with meshes directly extracted from confocal microscopy images of the mature onion adaxial epidermis demonstrate how guard cell opening can be achieved with an isotropic material. Experimental meshes shown with their originating z‐stacks from (a) paradermal view and (b) transverse section. Performing FEM simulations with these meshes resulted in an increase in pore area on inflation, without the need for geometrical constraints or complex cell wall properties (c). Pore area increase is independent of the starting pore area (d). Differently shaped icons in plots indicate meshes from different samples. Changes to the stiffness of the cell wall do not qualitatively impact the results, although introducing circumferential stiffening (anisotropy – dark magenta circles) increases the resulting pore area (e).

It has been previously shown that cell wall characteristics, such as material anisotropy in the form of circumferentially oriented microfibrils, strain‐stiffening and polar pinning, play important roles in stomatal opening (Carter *et al*., 2017; Woolfenden *et al*., 2017) in Arabidopsis. In particular, circumferential stiffness has been considered essential for FEM models to capture an increase in pore area upon guard cell inflation. These conclusions, however, were based on an idealised guard cell geometry in which the stoma was represented as a hollow, deformed torus with a circular cross‐section, and with solid end walls separating the two guard cells (Woolfenden *et al*., 2017). Contrary to our expectations, simulations using meshes derived directly from confocal microscopy (experimental meshes) readily and robustly reproduced pore opening without requiring any of these additional features. Results are shown in Fig. 1(c,d) for three exemplar samples, with mean data for all the experimental meshes used in this study shown in Fig. S1. These results were shown to be qualitatively robust across a range of different cell wall properties (Young's modulus, Fig. 1e). Moreover, the relationship between pore area and pressure followed the characteristic non‐linear curve, as described in Franks *et al*. (2001). In earlier modelling work using idealised geometry, we attributed this behaviour to strain‐stiffening (Woolfenden *et al*., 2017), yet here the same saturating response emerges even when using a simple isotropic material model with constant elasticity (Fig. 1c). The response observed was independent of the starting pore area (Fig. 1d), suggesting that the observed behaviour is linked to the starting geometry rather than the specific size of the guard cells.

Although the geometries of the experimental meshes have much in common, they also exhibited differences, such as individual irregularities, some arising from underlying structural features, including the influence of overlying cell wall junctions on segmentation, and others from imaging artefacts. To test the influence of these geometric variations, we developed simplified, idealised meshes to which we could add specific features observed in the experimental meshes, allowing us to test their influence on stomatal opening one at a time (see Methods S2). The impact of each characteristic was evaluated by observing whether it resulted in an increase or decrease in pore area upon guard cell inflation. To do this we used the approach described in Carter *et al*. (2017) and Woolfenden *et al*. (2017), of an initial standard torus mesh with a circular cross‐section. To enable comparison between the idealised and experimental meshes, we ensured consistency of pore area, pore length, stomatal length, and stomatal width, removing potentially confounding variables.

We found that idealised guard cell models represented as tori with initially circular guard cell cross‐sections and isotropic material predict stomatal closure on inflation (orange points in Fig. 2a), consistent with previous work (Carter *et al*., 2017; Woolfenden *et al*., 2017). In these idealised models, increasing turgor pressure causes the guard cells to bulge in width, which narrows the pore, rather than opening it. In sharp contrast, the experimental meshes capturing more realistic geometry predominantly exhibited pore opening when inflated under identical material properties and pressures (pink points in Fig. 2a). In addition, both the experimental meshes and idealised meshes with an initially oval cross‐section exhibit similar spatial patterns of surface stress and strain. In both cases, stress and strain are lower towards the outer stomatal wall and higher around the pore. This indicates higher deformation near the pore region, where it is most effective in driving aperture opening. The only difference between these idealised meshes and the experimental meshes lies in their initial geometry, as the material properties and wall thickness were kept the same. We therefore conclude that the idealised toroidal geometry fails to capture key geometric features necessary for stomatal opening mechanics.

**Fig. 2:**
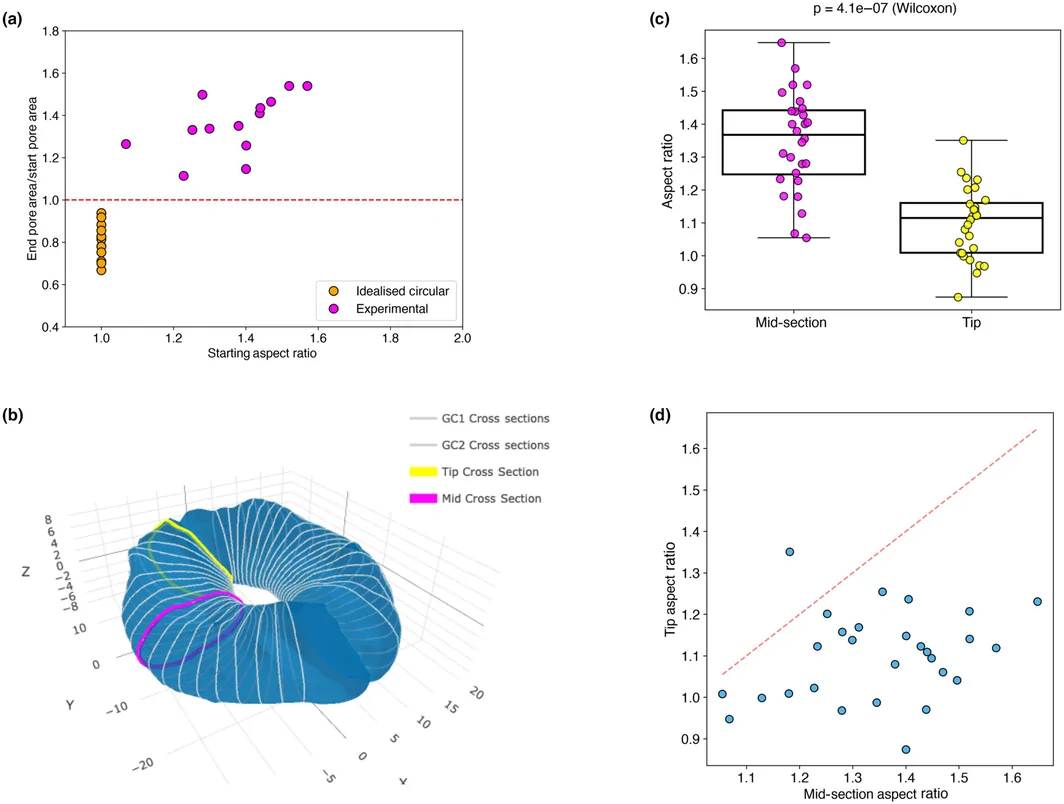
Onion stomata have a complex geometry, with distinct characteristics. Idealised meshes with circular cross‐sections show a decrease in pore aperture upon inflation, in contrast to the increase shown when simulating inflation of experimental meshes (a). Estimates of the cross‐sectional aspect ratio can be obtained by taking regular cross‐sections perpendicular to the centreline (corresponding to the minor circumference) along the length of the guard cell (b). Cross‐sectional aspect ratio at the midsection vs the tip when closed (low pressure). Box plots show the interquartile range (box) and median (line), with whiskers extending to 1.5× the interquartile range; individual data points are shown as jittered points. Wilcoxon signed‐rank test: *P* = 4.1e‐07 (c). Under these conditions, the aspect ratio at the tips is close to 1 (circular), with no correlation with the midsection aspect ratio (d).

### Cross‐sectional aspect ratio drives geometry‐derived stomatal opening

One key difference between a standard torus and our experimental meshes is their initial guard cell cross‐sectional shape. As shown in Fig. 2(b), the experimentally observed onion guard cells do not have an initially circular cross‐section; instead, they appear distinctly oval at the midpoint. To quantify this, regular cross‐sections were taken through experimental guard cell meshes, perpendicular to the centreline (Figs S2, S3), and the aspect ratio at each position calculated (Fig. 2b, compare tip (yellow) and mid‐sections (pink)). Cross‐sections taken at the guard cell midpoint had aspect ratios (width/depth) with a mean value of over 1.3, indicating an oval shape with the long axis in the plane of the surrounding epidermis (Fig. 2c). By contrast, the aspect ratio of cross‐sections taken towards the tips of guard cells was closer to 1 (mean value 1.1), indicating a rounder shape (Fig. 2b,c). There was no overt correlation of tip cross‐section shape with that at the mid‐section (Fig. 2d), suggesting that the pattern observed reflected a general shift towards a more oval shape in the mid‐section of all guard cells. The cross‐sectional shapes were similar across all meshes (Fig. S4). The cross‐sectional aspect ratio decreased approximately linearly along the length, reaching a minimum at *c*. 80% of the distance from the midpoint to the tip (Fig. S5).

To test the effect of initial guard cell cross‐sectional shape on stomatal function, we measured the cross‐sectional aspect ratio at the midpoint for each experimental mesh, then imposed this empirically measured aspect ratio along the entire length of the guard cell of a corresponding idealised mesh based on each guard cell image. Fig. 3a(i) shows an exemplar experimental mesh midpoint cross‐section (pink) from which an idealised oval (green) cross‐section has been obtained, then used to generate a guard cell shape. Fig. 3(a(ii,iii)) shows the calculated patterns of stress and strain, respectively, as the turgor pressure of the idealised mesh with an initially oval guard cell cross‐section in Fig. 3(a(i)) is increased. The calculated relative change in pore area for each of the idealised meshes with an initially oval guard cell cross‐section is shown in Fig. 3(d) (green dots). These data show that, with one exception, all modelled stomata consisting of guard cells with idealised oval mid‐cross sections opened (end pore area was larger than start pore area), with a positive correlation between the relative increase in pore area and starting guard cell cross‐sectional aspect ratio. By contrast, when an idealised circular cross‐section was derived from each experimental dataset (Fig. 3b(i)), all of the modelled stomata showed a decrease in pore size after increased turgor pressure (Fig. 3d, orange dots). This altered response relative to the idealised mesh with an initially oval guard cell cross‐section was also reflected in altered patterns of stress and strain (Fig. 3b(ii,iii), respectively). Whereas the idealised oval and experimentally derived geometries both concentrated stress and strain adjacent to the pore, the circular cross‐section produced a much flatter distribution, with strain of comparable magnitude spread evenly across the guard cell surface and the stress maximum lying over the outer wall rather than at the pore. Deformation was therefore not localised where it can act on the aperture, consistent with previous observations of uniform inflation increasing guard cell width and narrowing the pore. For comparison, Fig. 3c(i) shows the same initial experimentally derived guard cell midpoint cross‐section (as in Fig. 3a(i),b(i), pink circumference) but with the final cross‐sectional shape shown after guard cell inflation (brown line). The final guard cell cross‐sections of the experimental mesh samples are circular. Interestingly, the increase in length of the minor axis of the cross‐sectional shape (corresponding to guard cell height) is accompanied by a shortening of the longer axis (corresponding to guard cell width) of the original cross‐sectional shape (the brown circumference lies within the pink circumference). Thus, as the guard cell swells there is an increase in depth of the guard cell, but the width actually decreases (see Fig. S6), in line with the ‘archetypal Adiantum‐type guard cell deformation’ shown by Franks & Farquhar (2007). The inflation of the experimental mesh guard cells is accompanied by a distinct pattern of stress and strain (Fig. 3c(ii,iii)). When the relative pore area increase is calculated for the experimentally‐informed model, there is an increase in all samples which correlates with the starting guard cell cross‐section aspect ratio, with the relative increase in pore area being larger than that calculated for the idealised oval shaped guard cells (compare pink to green points in Fig. 3d).

**Fig. 3:**
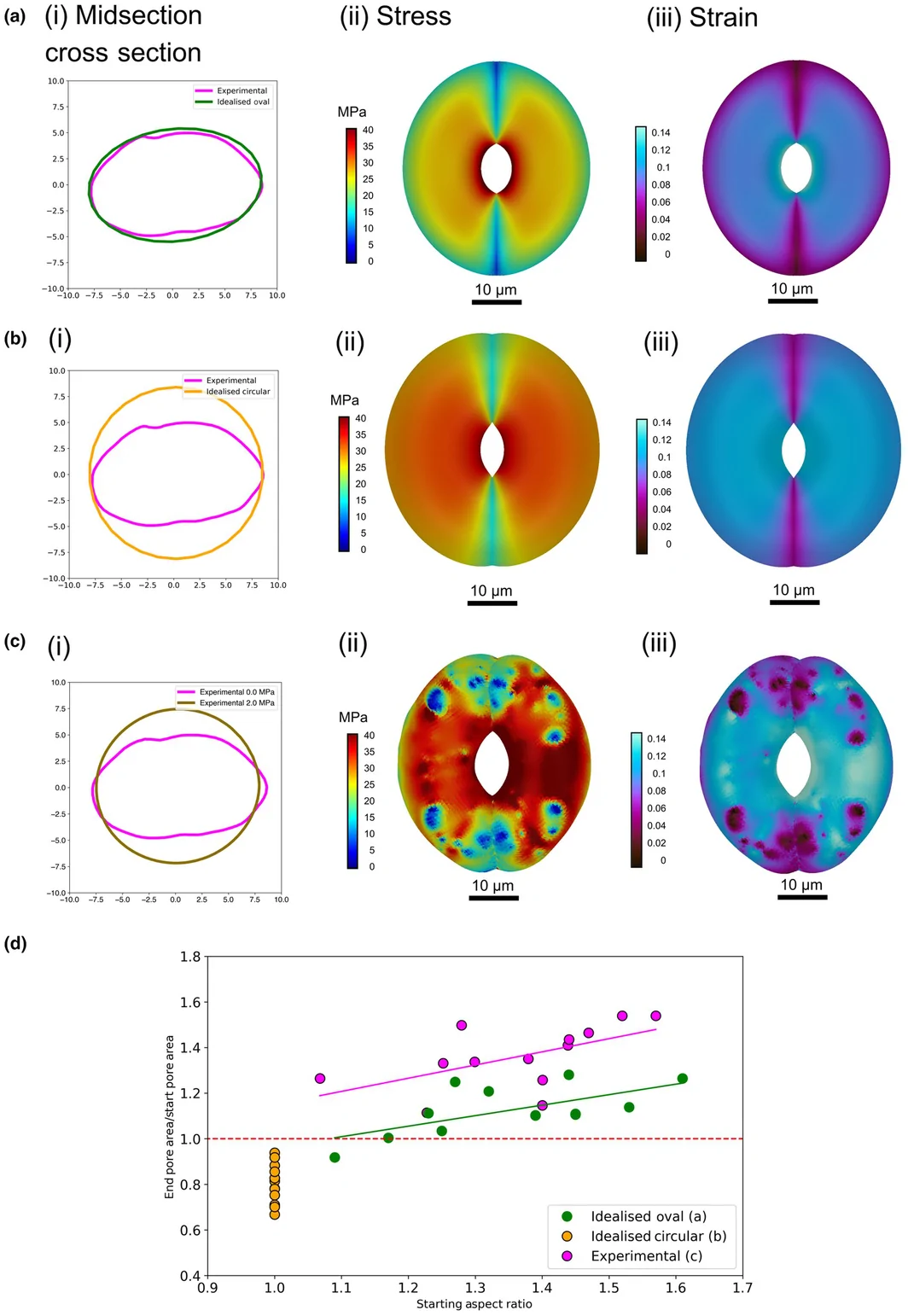
Guard cell geometry influences onion stomatal function. Finite element method (FEM) simulations were implemented using idealised meshes in which guard cell cross‐section was circular throughout (orange in b), an oval throughout (green in a) or based on experimentally observed cross‐sectional shape (pink in a–c). a(ii)–c(ii) show the stress patterns obtained when the meshes in a–c are inflated, where colour is the trace of the stress tensor, with the respective patterns of strain shown in a(iii)–c(iii). In (c), the experimentally observed guard cell cross‐section after inflation is shown in brown. (d) shows the relationship of the ratio of end pore area to starting pore area plotted against starting aspect ratio for simulations using the idealised circular cross‐sectional guard cell geometry (orange), idealised oval cross‐sectional guard cell geometry (green) and experimentally derived cross‐sectional guard cell geometry (pink). There is no relationship for the circular cross‐section data, but a positive correlation between aspect ratio and increase in pore area is observed for the oval data (Pearson *r* = 0.646, *P*‐value = 0.0171), and empirical data (Pearson *r* = 0.568, *P*‐value = 0.0427). The FEM models for all meshes have a simulated pressure increase of 2 MPa and have isotropic material properties. Points above the red dashed line in (d) show simulations for which inflation resulted in an increase in pore area (stomatal opening), whereas points below the line represent a decrease in pore area with inflation (stomatal closing). Simulations are shown in Supporting Information Video S1.

### Circumferential stiffness enhances, but is not essential for, stomatal opening

Up to this point, the wall material has been modelled as a uniform, isotropic material. However, the wall around guard cells is thought to be anisotropic, reflecting the orientation of microfibrils around the cell circumference (Shtein *et al*., 2017). To investigate the influence of such circumferential anisotropy on guard cell function, we introduced anisotropic wall stiffness into our experimentally‐based FEM meshes by increasing Young's modulus 10‐fold (E2 = 1000 MPa) in the circumferential direction relative to the longitudinal direction. This is shown diagrammatically in Fig. 4(a,b).

**Fig. 4:**
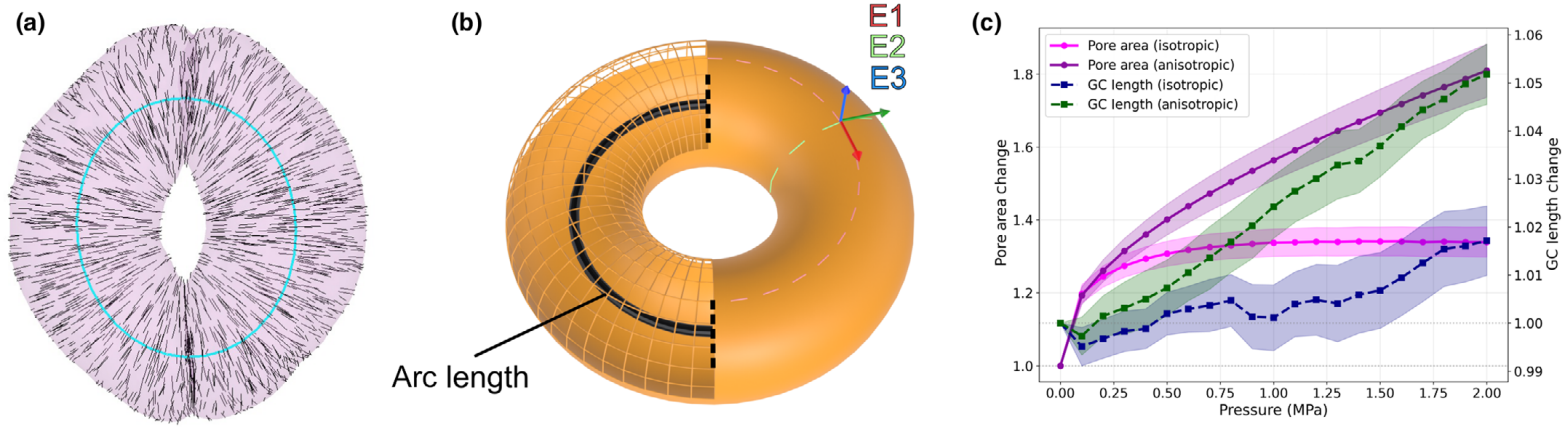
Anisotropic guard cell wall stiffness enhances onion stomatal pore opening by increasing guard cell arc length. (a) Visualisation of the material direction of increased stiffness (E2: grey lines) around an experimental mesh in MorphomechanX. (b) Cartoon representation of a guard cell pair with idealised circular cross‐section throughout to demonstrate the orthogonal vectors of modulus implemented in the model as well as the guard cell arc length. (c) Anisotropic stiffening, resulting from circumferential orientation of cellulose microfibrils, was implemented by an increased (10×) Young's modulus in the E2 modulus. This acts to restrict radial expansion, thus promoting longitudinal lengthening. Experimental meshes with anisotropic stiffness (purple line) show greater increases in pore area with pressure than meshes with isotropic properties (pink line). There is also an increase in guard cell arc length in the anisotropic models (green line) compared with the isotropic samples (blue line). Values shown are means (*n* = 14) and the shaded sections show the standard error (SE).

Considering first pore area response (Fig. 4c), at low pressure increases (< 0.25 MPa), circumferential stiffness produced no meaningful enhancement of pore opening compared with the isotropic experimental meshes (pink and purple lines coincide). However, at higher pressures, circumferential stiffness resulted in substantially greater pore area increases (pink line reaches an asymptote while purple line shows a more linear response to pressure), consistent with previous modelling predictions (Woolfenden *et al*., 2017). This suggests that geometry is the main determinant of stomatal function at lower pressures, whereas at higher pressures circumferential stiffness allows for greater overall pore apertures. To test the hypothesis that the increased pore area in models with circumferential stiffness arises from increased longitudinal stretching of the guard cells, we measured the change in guard cell arc length, defined as the curved line running through the middle of the cross‐section, under inflation (Fig. 4b). We found that the models with circumferential stiffness showed greater arc length increases than isotropic models (compare green and blue lines in Fig. 4c), with the divergence in arc length coinciding with the divergence in pore area observed at higher pressures (Fig. 4c). Stress distributions were comparable between the isotropic and anisotropic cases, with maxima on the ventral wall adjacent to the pore and minima at the polar junctions in both, suggesting that geometry and turgor, rather than wall material properties, are the main determinants (Fig. S7a,c). By contrast, strain distributions differed markedly between the two cases. Under isotropic stiffness, strain was high and approximately uniform across the guard cell surface and showed no strong directional bias. In the anisotropic case, strain was low over most of the wall and concentrated around the ventral wall and at the polar junctions, and was predominantly longitudinal (Fig. S7b,d). Anisotropy therefore redirects cross‐sectional inflation – which displaces the ventral wall into the pore and reduces pore‐area gain – into extension along the long axis of the guard cell. Combining these observations (Fig. 4c) revealed that in models based on the experimentally derived guard cell cross‐sectional shape and incorporating circumferential anisotropic stiffness, there is an initial relatively rapid increase in pore area at lower turgor pressure (reflecting the geometry‐driven changes in guard cell cross‐sectional shape), followed by a slower but prolonged increase in pore area (driven by the increase in guard cell arc length). This suggests that stomatal opening is a two‐phase process.

## Discussion

Numerous models of stomatal mechanics have been proposed over the years (Guttenberg, 1959; Woolfenden *et al*., 2018; Tan *et al*., 2025). In most of these models, a role for circumferential anisotropic stiffness (resulting from the observed preferred orientation of cellulose microfibrils) has been implemented and suggested to be essential for stomatal function (effective opening and closing of the stomatal pore). Contrary to these previous models, we find that circumferential stiffness is not required for stomatal opening. This advance was enabled by our use of confocal images to directly inform FEM simulations, capturing and implementing a guard cell geometry much closer to reality than in most previous models. In addition to circumferential anisotropy, differences in cell wall thickness along the guard cell wall have also been proposed to contribute to stomatal mechanics. However, unlike the systems considered in those studies, onion guard cells have increased cell wall thickness at their basal region rather than in the region adjacent to the pore (Fig. S8; Table S1). We tested the effect of introducing increased basal wall thickness in our model and found that this does not qualitatively alter the deformation behaviour; within our framework, such thickness variations modulate the magnitude of deformation rather than introducing distinct modes of opening (Fig. S9). The lack of dependence on circumferential stiffness may at first sight seem surprising given the ubiquity of its presence; however, there are data in the literature that have questioned its importance. For example, recent work by Keynia *et al*. (2023) examining a range of cell wall mutants revealed that some level of stomatal function was still present in plants where cell wall structure (including cellulose) was significantly altered, suggesting that guard cells could somehow compensate for changes in wall material properties. Guard cell geometry might be at least part of the mechanism of compensation underlying these observations. It is important to note that while our modelling does indicate that the presence of circumferential stiffness is beneficial to stomatal pore opening, it is not as essential as previously believed. This nuance has potentially important implications for our understanding of stomatal evolution.

Our simulations on experimentally derived meshes show a robust increase in pore area under increasing turgor pressure without any special requirements for wall material properties, such as anisotropy or strain‐stiffening. This behaviour is mirrored in idealised meshes when the experimentally measured guard cell cross‐sectional aspect ratios are applied. These results support the hypothesis that the specific geometry of the guard cells is sufficient to underpin observed stomatal opening. The shape changes implied by our model revolve around the circularisation of the guard cell's cross‐section, which causes a simultaneous increase in height, and importantly for pore width, a narrowing of guard cell width (see Fig. S6). This process can be compared to that previously observed in the mechanism of explosive seed dispersal where a change from cell widening to shortening creates the potential energy required for eventual rapid release of kinetic energy (Hofhuis *et al*., 2016; Mosca *et al*., 2024). Both systems highlight how active control of cell shape change can underpin important processes in plant biology, adding to a growing realisation of the importance of geometry in biological systems. The impact of cross‐sectional shape change on gas exchange also depends on the inversely proportional relationship between guard cell depth and stomatal conductance (Franks & Beerling, 2009). Using a fixed pore diffusion model (Parlange & Waggoner, 1970), we found that under isotropic material properties, the increase in conductance from larger pore area is partly offset (*c*. 64%) by increased guard cell depth. By contrast, anisotropic stiffness increases pore area without a corresponding increase in guard cell depth, reducing this offset to *c*. 17% and yielding a greater net conductance gain (Fig. S10). However, this diffusion model assumes that the greater guard cell depth increases the path for diffusion to the substomatal cavity. This assumption does not fully capture the geometry of onion stomata, where guard‐cell expansion occurs predominantly into the substomatal cavity. Thus, an increase in guard‐cell depth does not necessarily correspond to an equivalent increase in the diffusion path, and the predicted reduction in conductance may therefore be overestimated in onion. Nevertheless, this highlights that stomatal efficiency depends not only on total pore area, but also on how the geometry affects rates of gas exchange (Franks & Farquhar, 2007; Franks & Beerling, 2009).

Once the guard cells have reached a maximal mid‐section cross‐sectional area (circular), further inflation leads to lengthening of the cells, a process that is maximised by the presence of circumferential anisotropy, albeit at the cost of requiring higher turgor pressure. The geometry‐dependent nature of stomatal opening has been recognised for many decades. Paton & Pearce (1957) described a comparable mechanism in bryophyte stomata, in which changes in guard‐cell shape during inflation contribute to pore opening, while subsequent work by Sharpe & Wu (1978) and Wu *et al*. (1985) developed a quantitative mechanical interpretation of the relationship between guard cell geometry and stomatal opening. More recently, Bidhendi & Geitmann (2018) proposed that the nonlinear pressure‐aperture behaviour observed experimentally could also arise from a two‐step opening mechanism: an initial turgor‐induced change in guard cell geometry from elliptical to circular (inflation), followed by cell wall stretching upon further inflation. Our results demonstrate how these geometry‐dependent mechanisms can give rise to the previously observed ‘biphasic’ behaviour of stomata (Stålfelt, 1927). Rather than reflecting a single mechanism operating at different rates, the behaviour arises from the combination of two mechanisms, with the first dominating early in opening, and the second becoming increasingly important during later stages. Although both stages involve pore opening and therefore fall within Stålfelt's ‘Motorphase’ (Sharpe *et al*., 1987), our results reveal distinct mechanical contributions within this phase. Finally, it is worth noting that early FEM studies of guard cells also highlighted the importance of guard cell cross‐sectional geometry (Cooke *et al*., 1976), although the contour shape change in Cooke *et al*. (1976) depended on boundary conditions, this is not required in our present model. Although Manandhar *et al*. (2026) visualised significant mechanical interactions between guard cells and adjacent epidermal cells, the extent to which surrounding cells constrain guard cell deformation may vary with stomatal complex organisation and the spatial arrangement of neighbouring and subsidiary cells (Pautov *et al*., 2024). Our results indicate that, in onion, realistic guard cell geometry alone is sufficient to reproduce the observed deformation, without explicitly modelling neighbouring cells or imposing additional boundary constraints to represent their mechanical effects. Overall, our findings highlight the crucial role of geometric features in controlling stomatal dynamics and demonstrate that simple isotropic models, when geometrically accurate, can reproduce complex stomatal behaviour.

Our finding that anisotropic stiffness contributes to, but is not essential for, stomatal opening has implications for stomatal adaptation and evolution. Geometry alone is sufficient to enable pore opening at low turgor pressures whereas anisotropic stiffening becomes more important at higher pressures, allowing larger maximum apertures. From this viewpoint, the initiation of a functional stomatal structure at some point in evolutionary history would require only the formation of an appropriate proto‐guard cell shape around a pore to allow some advantage in gas flux control. Subsequent accretion of wall anisotropy would provide added potential advantage if an increased range of pore movement (thus gas flux) was advantageous. Taking this further, one can envisage a particular combination of guard cell geometry (e.g. guard cell cross‐sectional shape) and wall properties (e.g. anisotropy) placing a stomate at a point in morphospace which aligns the stomatal structure with physiological function (gas exchange capacity). In this scenario (Fig. 5), stomata in different species might achieve quite similar anatomically set physiological function by different combinations of guard cell geometry and wall material properties. Moreover, evolutionary (or even more short‐term) adaptation to particular environments might occur via movement through morphospace in a diverse set of pathways. For example, in the short term, there is good evidence that guard cell shape alters as stomata mature (Jaafar *et al*., 2024) and that this influences stomatal function. Our modelling platform provides a means to test this hypothesis. At the level of speciation, physiological data indicate a range of stomatal performance across broad evolutionary boundaries (Brench *et al*., 2025), providing a means to investigate to what extent such differences might relate to shifts in geometry and/or wall properties. For example, species that respond quickly to environmental fluctuations may tend to have stomata with higher cross‐sectional aspect ratios, while those optimised for maximal gas exchange may exhibit stronger circumferential stiffening. Quantifying the relative contributions of geometry and anisotropic stiffening in a range of species will allow testing of this hypothesis. We propose that future investigation of the interplay between geometry and wall properties across development and under varying environmental conditions in a range of species will be essential to fully understand stomatal mechanics and performance.

**Fig. 5:**
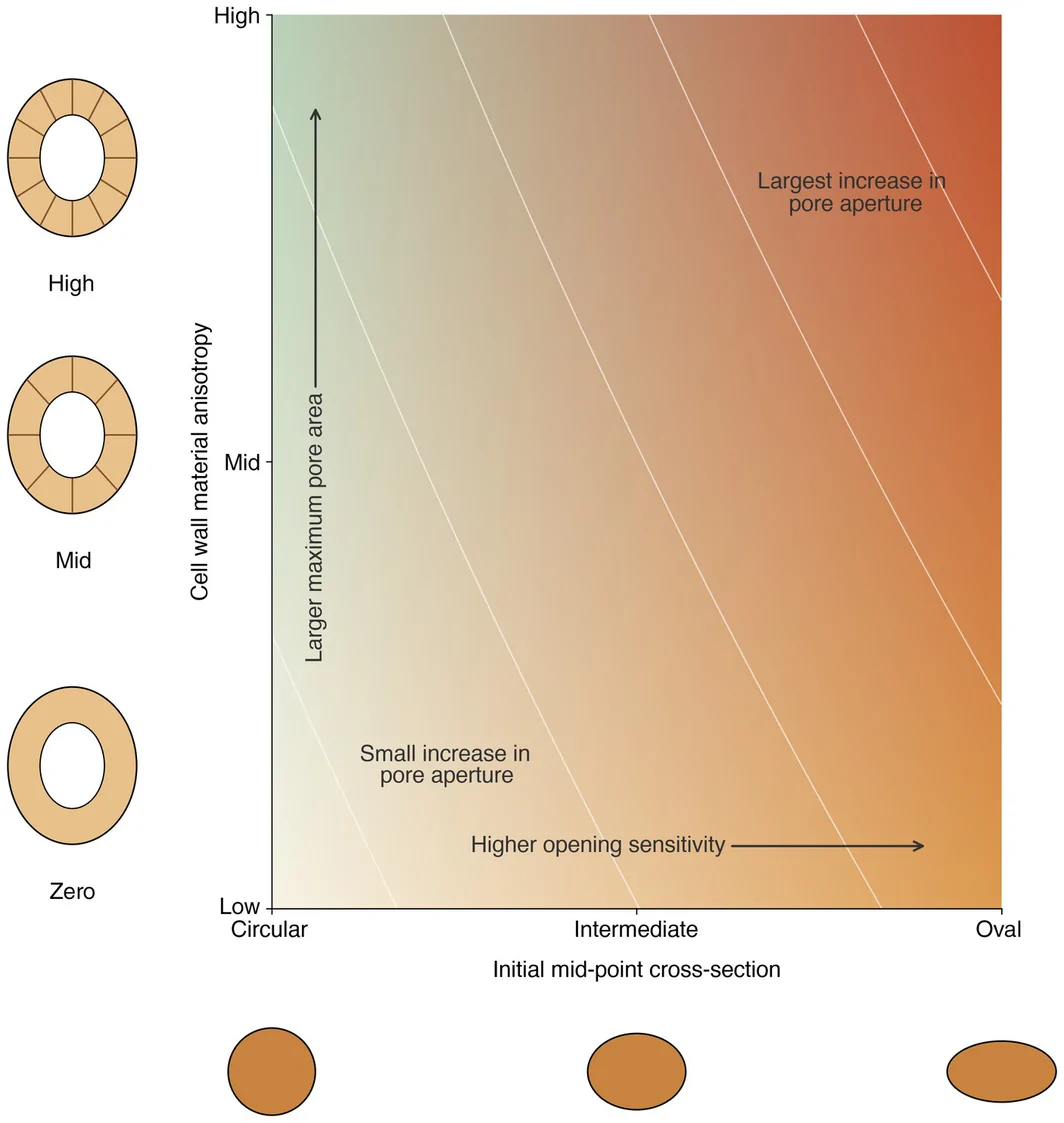
Conceptual framework for shifting onion guard cell shape (mid‐section aspect ratio) and wall material properties (anisotropy) to drive altered stomatal function. Moving along the *x*‐axis increases sensitivity to pressure change, whereas moving up the *y*‐axis increases the maximum range of potential pore area change. The colour intensity indicates maximal relative pore area change per unit pressure change. This colour range defines a potential landscape for stomatal form and function on which evolution could act.

## Competing interests

None declared.

## Author contributions

MT, RJM and RSS conceptualized the study. MT and RSS designed the methodology. MT and RSS involved in software development. MT and MJW validated the study. MT performed the formal analysis. MT and MJW investigated the study. BP, MJW and JA provided most of the resources. MT wrote the original draft. MT, RJM, RSS, MJW, JA, NYHT, AJF and JEG contributed to review and edit the article. MT and MJW visualized the manuscript. RJM, RSS, AJF and JEG supervised the study. RJM and AJF involved in project administration. RJM, RSS, AJF and JEG contributed to funding acquisition.

## Disclaimer

The New Phytologist Foundation remains neutral with regard to jurisdictional claims in maps and in any institutional affiliations.

## Supporting information


**Fig. S1.** Pressure–pore area relationship for all experimental meshes, with the mean highlighted.
**Fig. S2** Automated central‐line determination used to extract guard cell cross‐sections.
**Fig. S3** Cross‐sections taken along the length of an uninflated and inflated stomatal mesh.
**Fig. S4** Overlays of cross‐sections from all confocal meshes (*n* = 14), tip to mid‐section.
**Fig. S5** Cross‐sectional aspect ratio along the length of the guard cell (median and individual meshes).
**Fig. S6** Increase in guard cell height with a corresponding decrease in width upon inflation (a: experimental, b: idealised oval meshes).
**Fig. S7** Directions of stress and strain for finite element method simulations with isotropic and anisotropic cell wall stiffness.
**Fig. S8** Cross‐sections of closed stomata showing consistency of shape and wall thickness.
**Fig. S9** Simulation of increased basal wall thickness (2× and 4× the remaining guard cell wall).
**Fig. S10** Mean relative changes in stomatal conductance with pore area and pore depth (isotropic and anisotropic).
**Methods S1** Pipeline for automatically extracting features from meshes.
**Methods S2** Pipeline for creating the idealised meshes.
**Table S1** Measured non‐uniform guard cell wall thickness (lower wall ~3.04× thicker than upper).

**Video S1** Finite element method simulation of stomatal opening for experimental and idealised meshes.
Please note: Wiley is not responsible for the content or functionality of any Supporting Information supplied by the authors. Any queries (other than missing material) should be directed to the *New Phytologist* Central Office.

## Data Availability

All software, models and scripts to run the simulations are publicly available at https://zenodo.org/records/18955650. The scripts to process the data are publicly available at https://github.com/mtomtom/onion‐stomatal‐geometry. Original microscopy confocal stacks are available at https://zenodo.org/records/14793472.
