# Supplementary material for: Dual mechanisms give rise to biphasic stomatal opening in onion
Source: New Phytol. 2026 Oct 6;252(5):1976–87. doi: 10.1111/nph.71615 (PMC13738668; doi:10.1111/nph.71615)
Supplement: Supplementary file 1 [file NPH-252-1976-s002.pdf]

New Phytologist Supporting Information

Article title: Dual mechanisms give rise to biphasic stomatal opening in onion

Authors: Melissa Tomkins, Matthew J. Wilson, Jodie V. Armand, Nathanael Y. H. Tan, Briony Parker, Julie E. Gray, Andrew J. Fleming, Richard J. Morris and Richard S. Smith

Article acceptance date: 7 September 2026

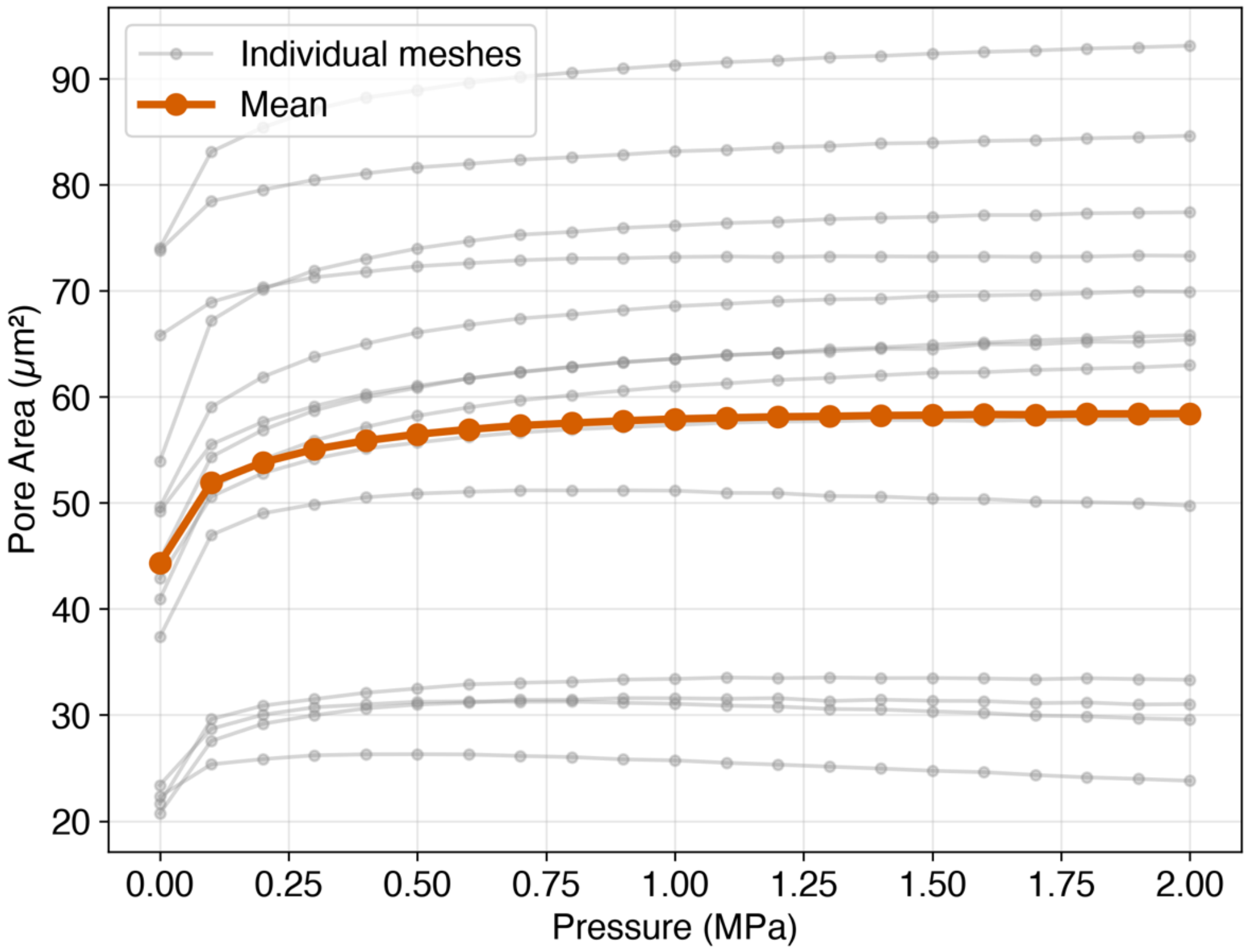

Fig. S1: The pressure vs. pore area relationship for all the experimental meshes used in this study. The mean value is highlighted in orange.

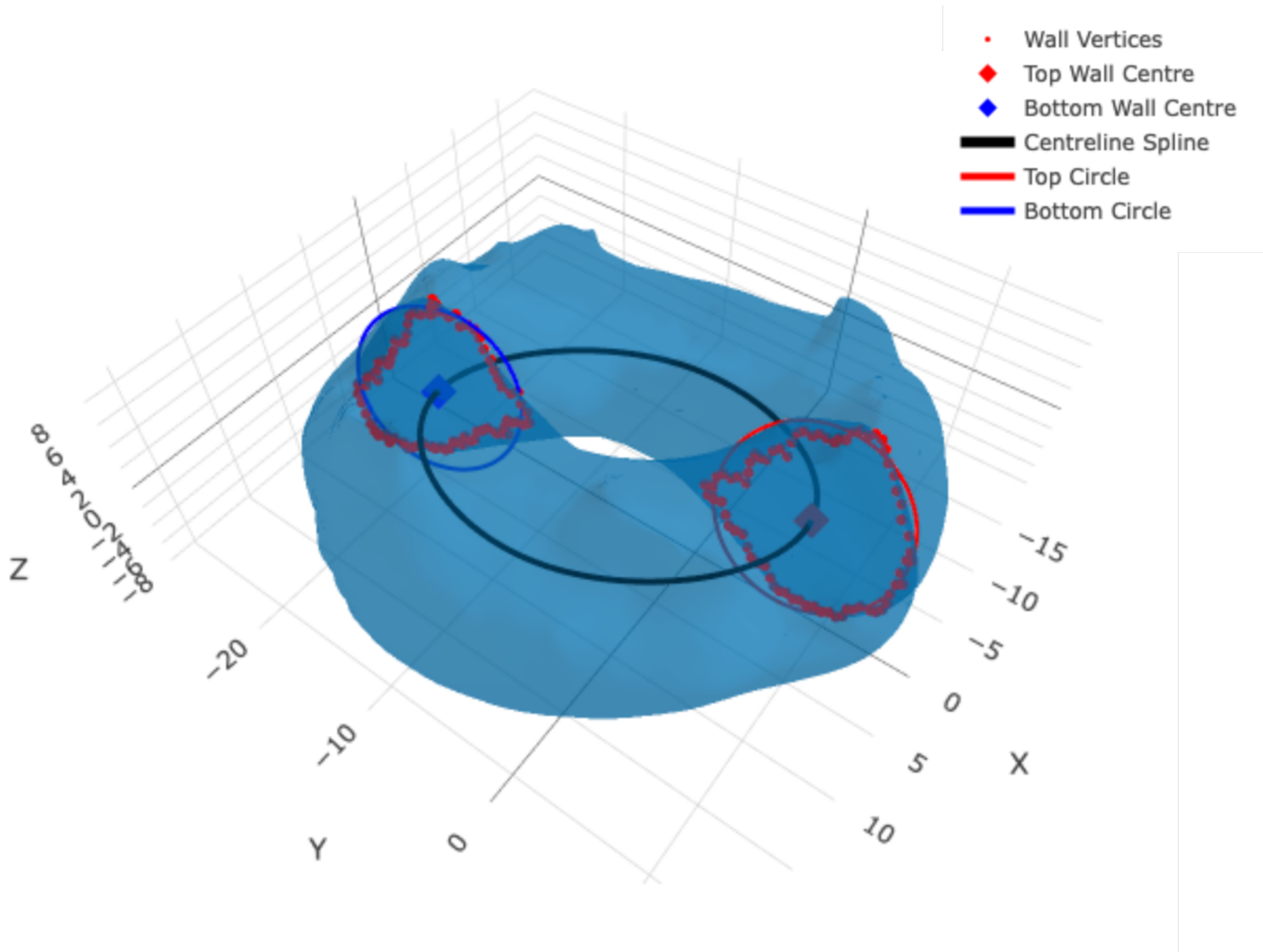

Fig. S2: The automated function (**Methods S1)** determines a central line, perpendicular to which the cross-sections are taken, based on an estimated central point of the guard cell – guard cell wall. The central point is estimated based on circles with an equal cross-section area to the midpoint of the guard cell.

a) b)

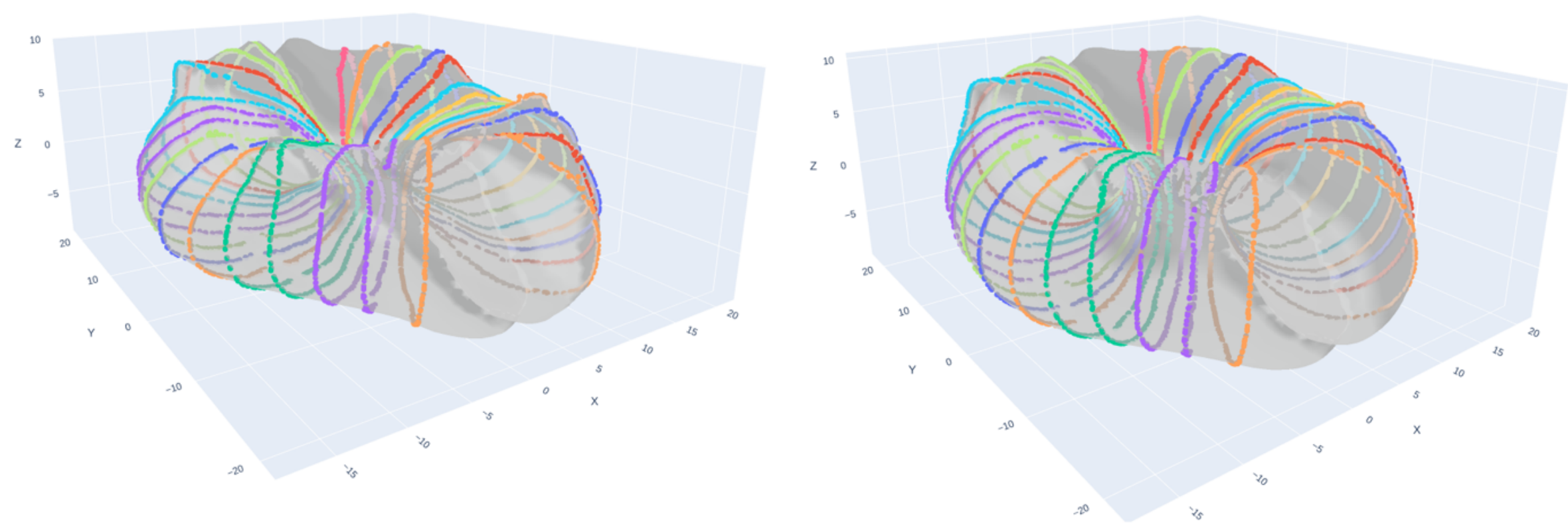

Fig. S3: The greatest change to the cross-sectional aspect ratio takes place at the midpoint, with there being little change nearer the tips, suggesting that the greatest deformation occurs at a point maximising impact to the increase in pore area. Cross-sections taken along the length of an uninflated (a) and inflated (b) stomatal mesh.

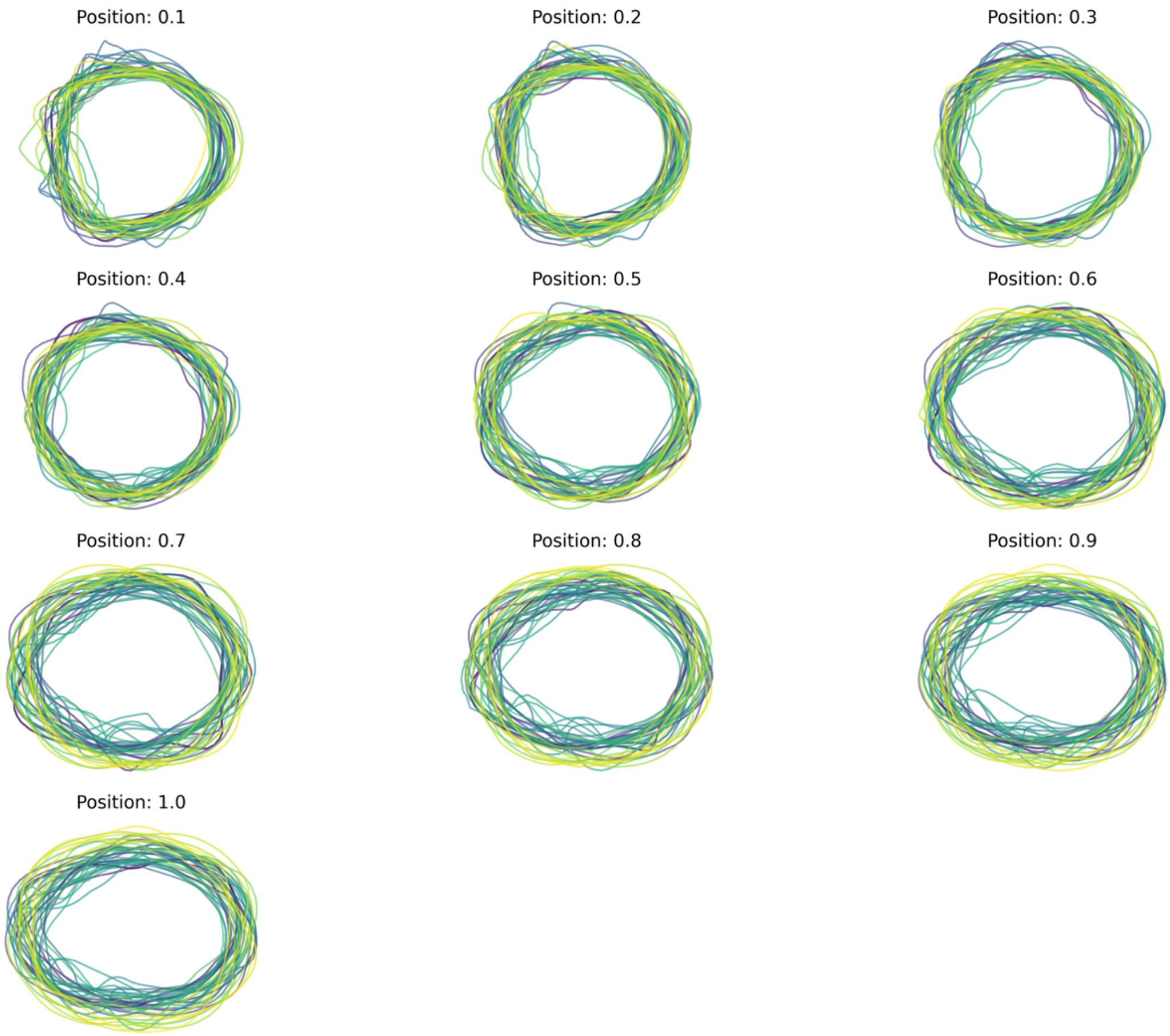

Fig. S4: Overlays from cross-sections from all confocal meshes (N = 14) taken at regular points between the tip (0) and the mid-section (1) show that the guard cells move from being circular at the tips to ellipsoid at the midpoints.

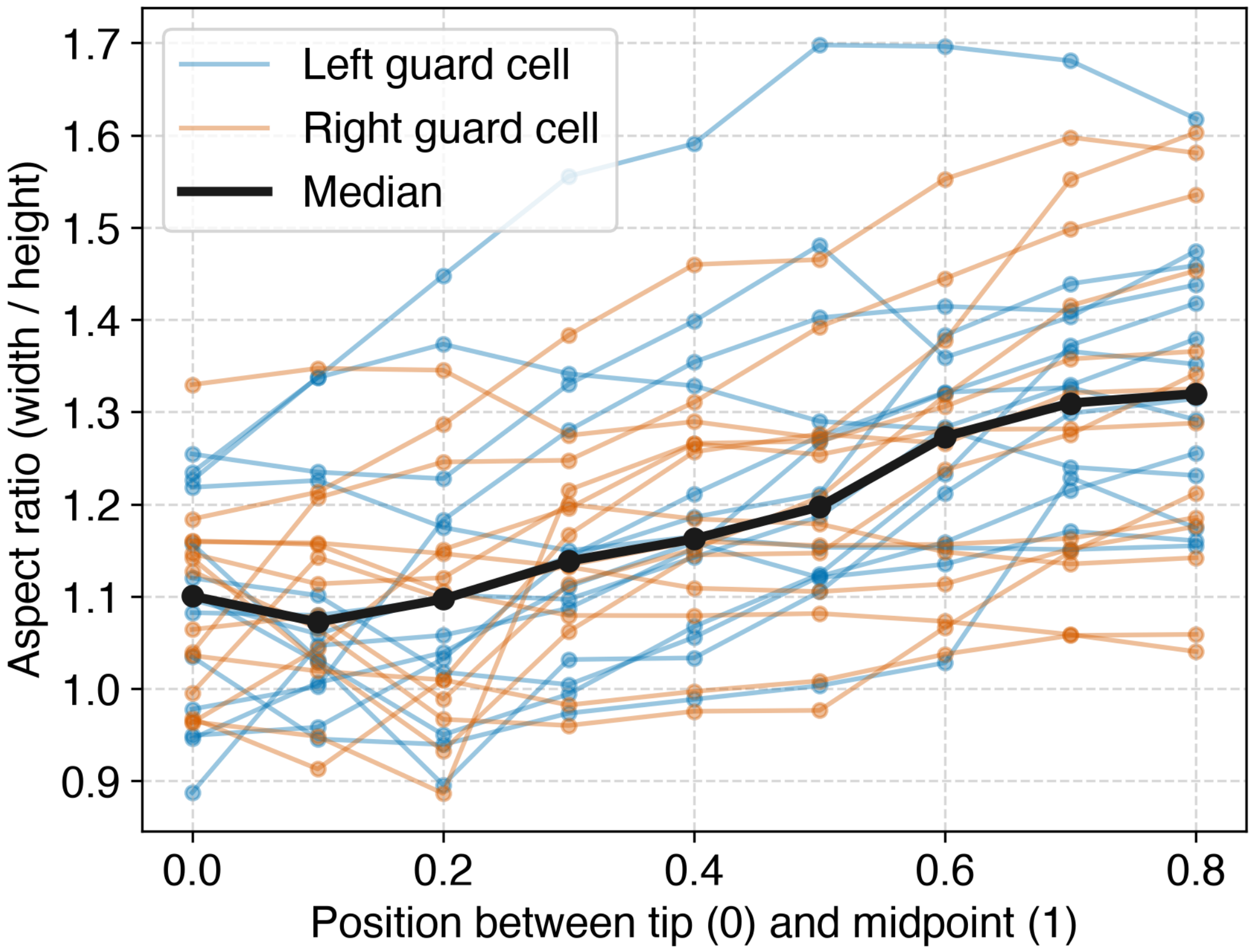

Fig. S5: Median values (black line) for the individual meshes (coloured lines) showing how the cross-section aspect ratio varies along the length of the guard cell.

a) Experimental meshes b) Idealised oval meshes

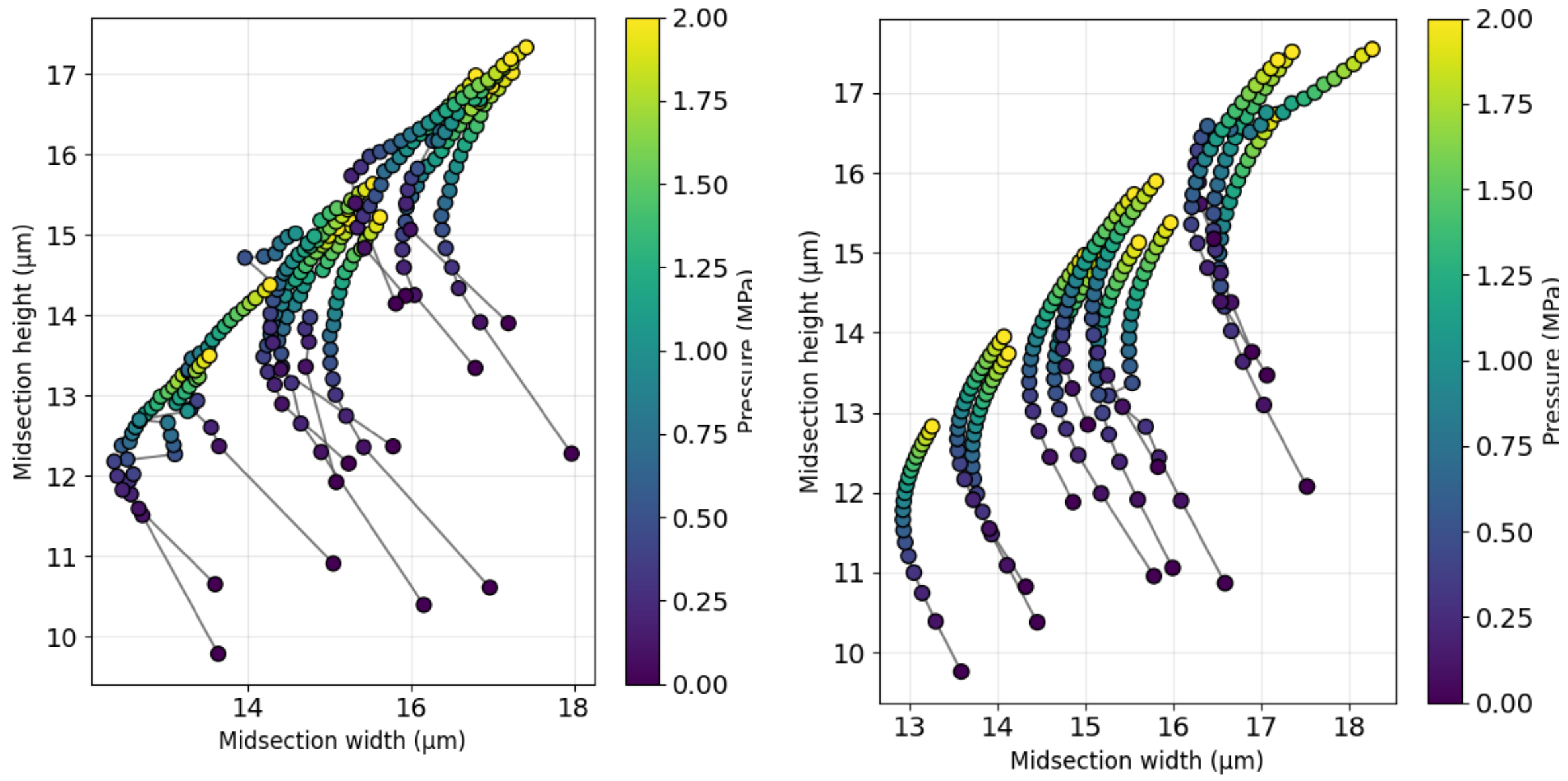

Fig. S6: Initial increases in pressure result in an increase in guard cell height, with a corresponding decrease in guard cell width for models run with both a) experimental and b) idealised oval meshes.

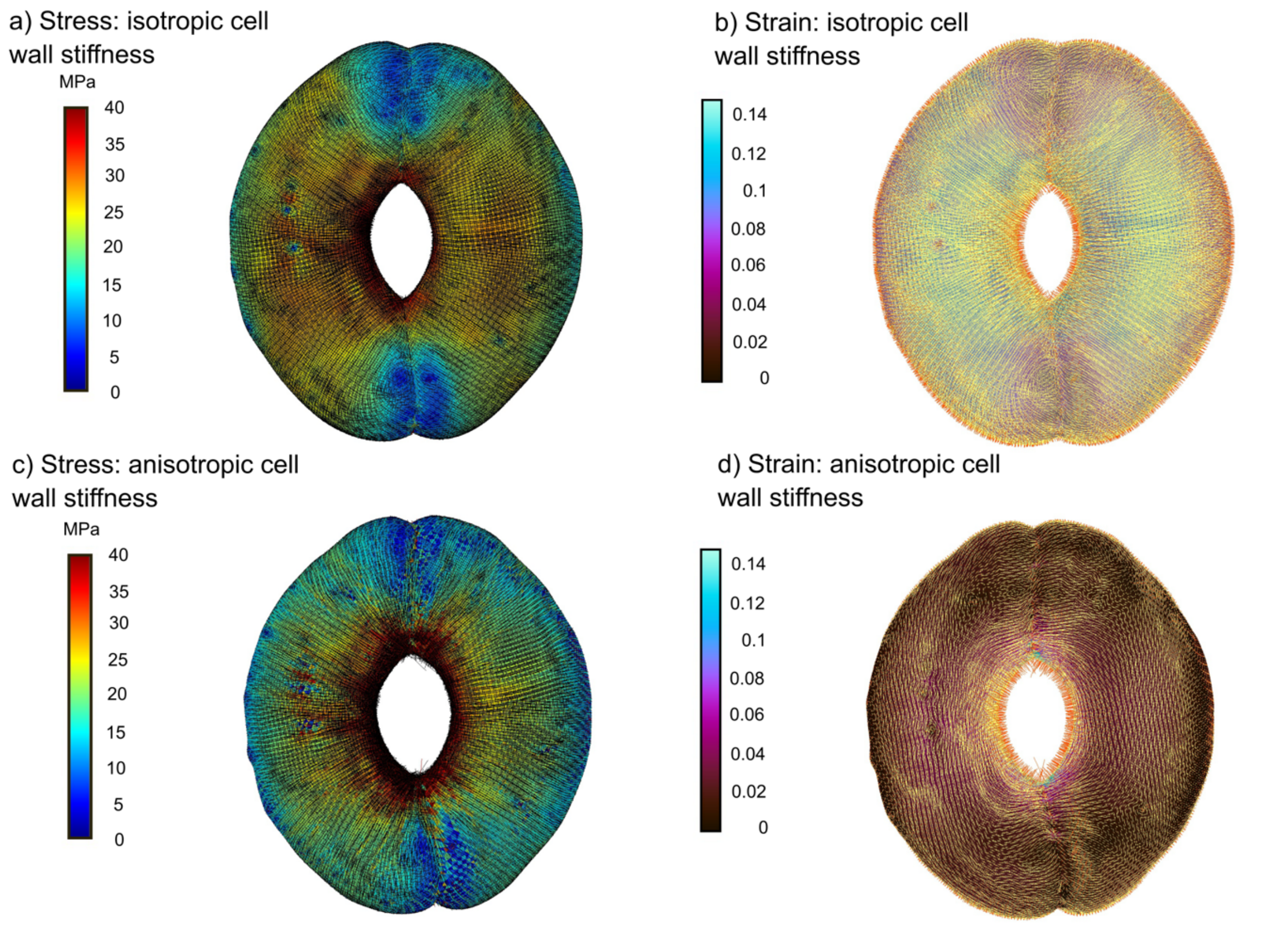

Fig. S7: Comparing directions of stress and strain for FEM simulations with isotropic (a, b) and anisotropic (c, d) cell wall stiffness. Directions of stress (black lines in a, c) are isotropic in both cases. Directions of strain (yellow lines in b, d) are isotropic when the cell wall material properties are isotropic, but longitudinal when the cell wall material properties are anisotropic, demonstrating that the guard cells increase in length, whilst being constrained in width.

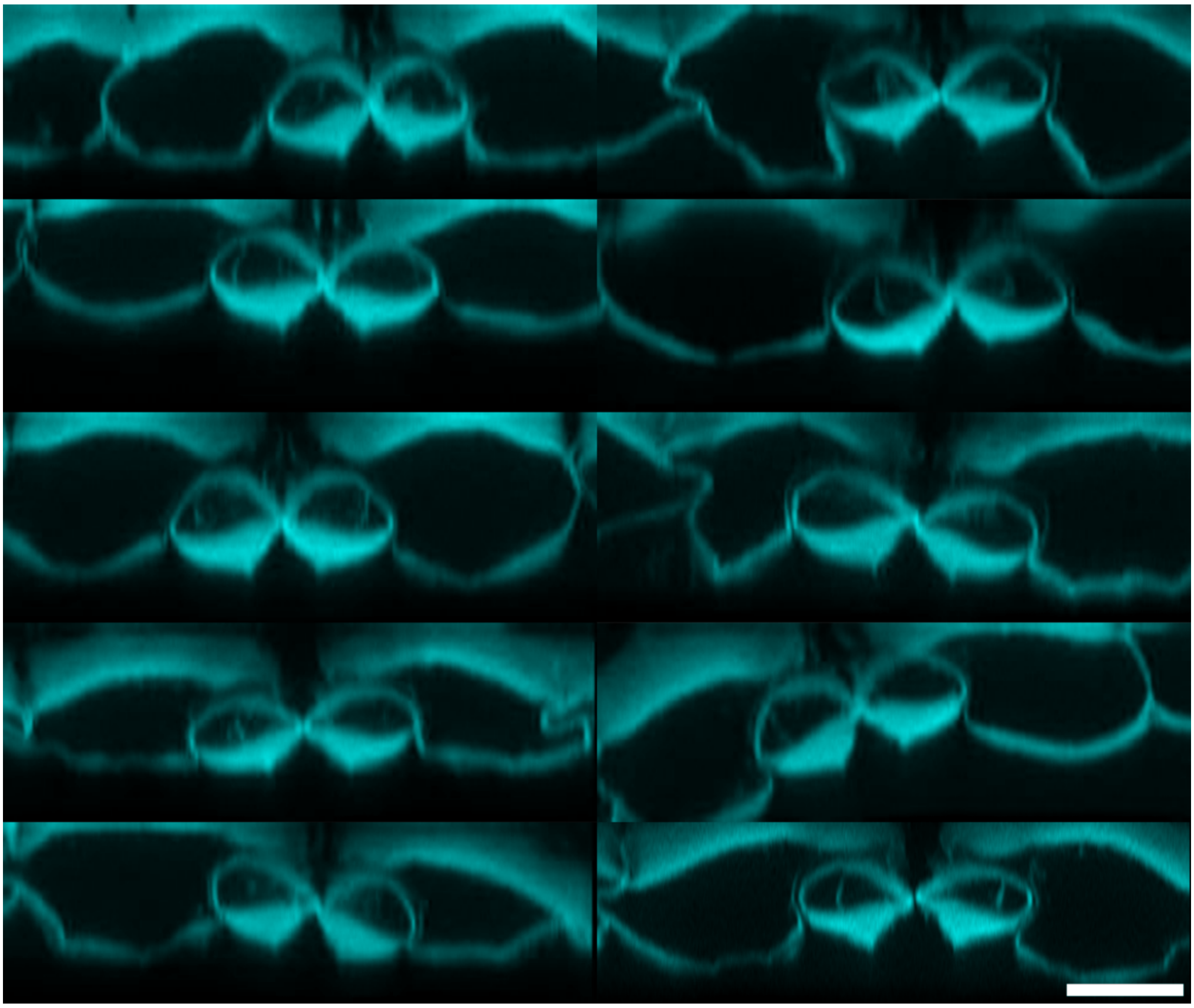

Fig. S8: Cross sections across a range of closed stomata used in this study, showing the consistency of shape and wall thickness.

Table S1: Onion guard cells have non-uniform wall thickness, with the lower (exposed portion) wall being, on average, 3.04x as thick as the upper (constrained) cell wall.

ID (Ac_DA_)	Lower GC1	Lower GC2	Lower GC mean	Upper GC1	Upper GC2	Upper GC mean	Ratio GC1	Ratio GC2	Ratio mean
1_2	7.82	7.93	7.88	1.94	1.74	1.84	4.04	4.55	4.29
1_3	7.84	8.10	7.97	2.80	2.64	2.72	2.80	3.07	2.93
1_4	8.31	8.01	8.16	2.63	2.28	2.46	3.16	3.51	3.33
1_5	8.85	7.90	8.38	4.16	2.92	3.54	2.13	2.70	2.42
1_6	7.90	7.86	7.88	2.52	2.51	2.52	3.13	3.13	3.13
1_8	7.28	7.00	7.14	2.87	1.39	2.13	2.54	5.05	3.79
2_1	7.37	7.40	7.39	2.80	2.91	2.85	2.63	2.55	2.59
2_3	8.40	8.11	8.26	3.88	3.11	3.50	2.16	2.61	2.38
2_7	7.13	6.43	6.79	2.29	3.11	2.70	3.12	2.07	2.59
3_2	8.65	9.19	8.92	3.39	2.86	3.12	2.55	3.22	2.88
3_4	9.96	9.52	9.74	3.29	3.69	3.49	3.03	2.58	2.81
3_6	8.53	8.45	8.49	2.84	3.67	3.25	3.00	2.30	2.65
3_7	9.35	9.39	9.37	2.60	2.36	2.48	3.59	3.99	3.79

We simulated an increased thickness by setting the parameter determining thickness to 2x and 4x the thickness of the rest of the guard cell wall (Fig. S9), and found that this had a minimal impact on pore opening for isotropic conditions: for mesh 1_2, increase in pore opening (end pore area / start pore area) was 1.54, 1.53 and 1.49 for increased thickness for the lower portion of the stomata for 1x, 2x, and 4x respectively. As would be expected, this had a greater impact on stomatal opening when applying anisotropic conditions, as the elongation of the guard cells would be reduced: 1.97, 1.84 and 1.71 for 1x, 2x and 4x respectively. To test that the uneven nature of the thickness had no impact, we also tested a uniform thickness of approximately (a slight overestimate as less than half of the guard cell wall had additional thickness applied) the average of the two increased thickness cases (1.5x, and 2.5x). Under the assumption of isotropy, we saw a pore increase of 1.52 and 1.47, and in the anisotropic case, a pore increase of 1.8 and 1.63. The closeness of these results show that, for this case, uneven wall thickness has no effect, with a single thickness parameter will give the same outcome.

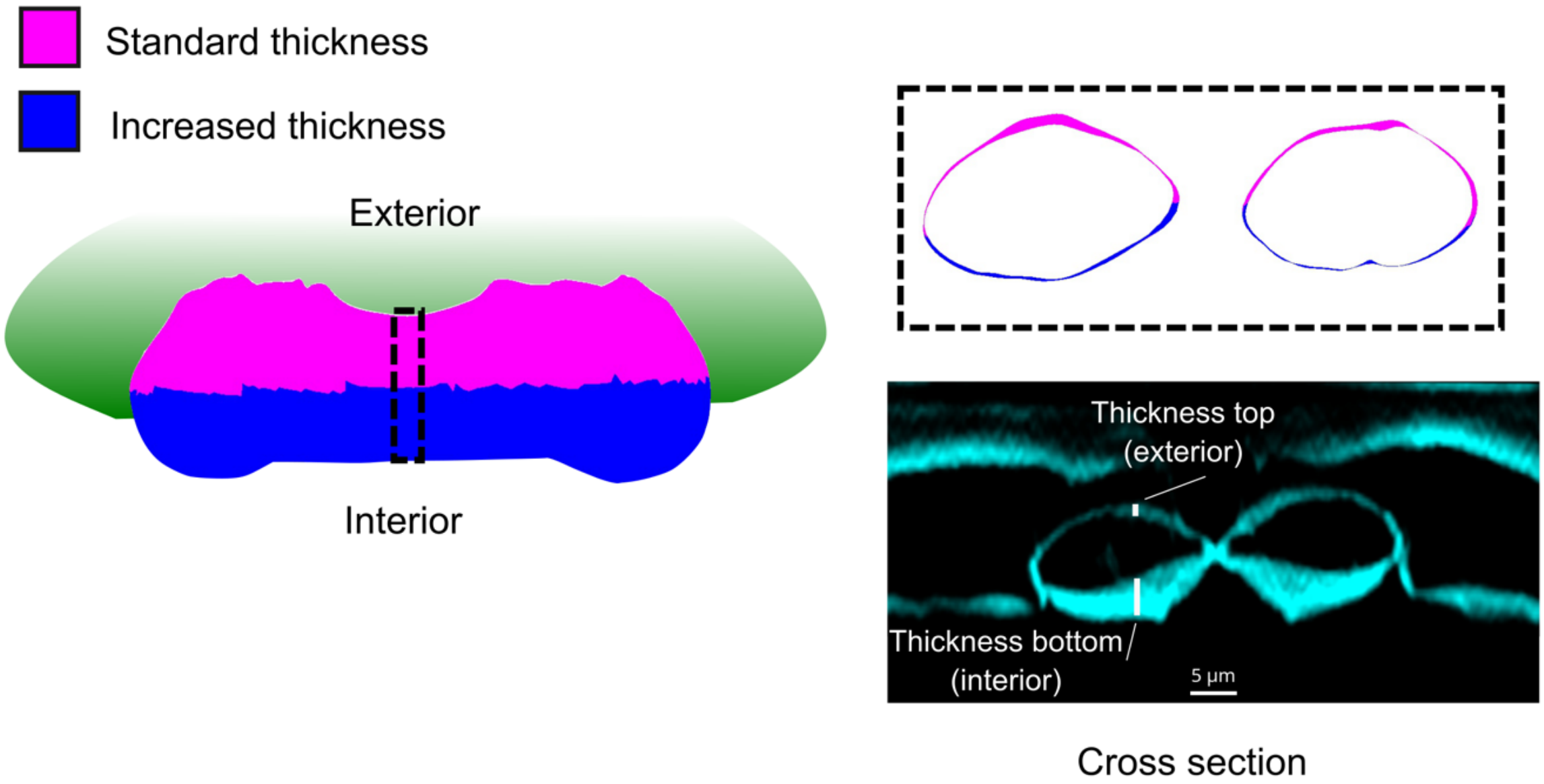

Fig. S9: To simulate thickness, the area corresponding to the thicker wall seen in the confocal images was parameterised to represent a thickness of 2x and 4x the thickness of the rest of the guard cell.

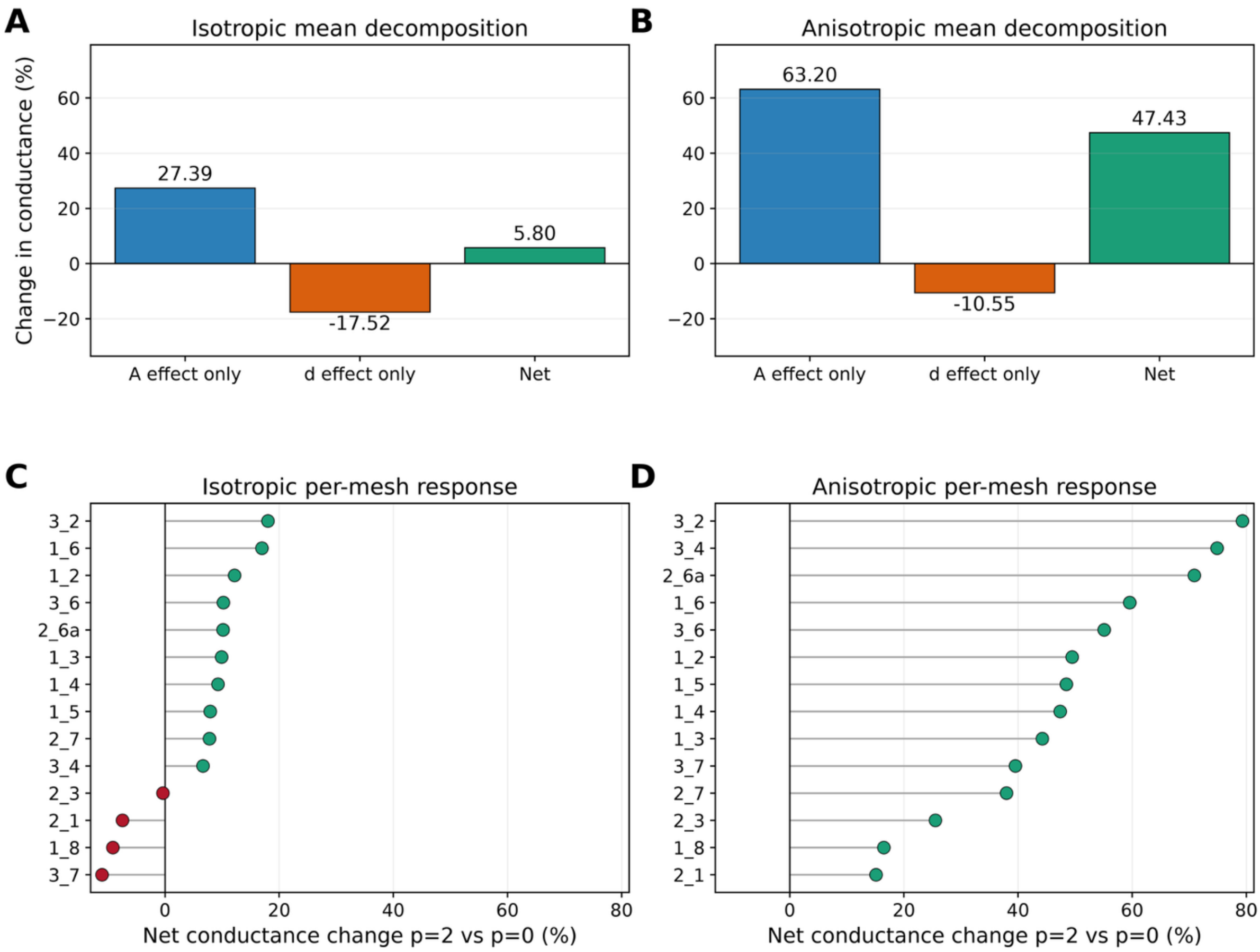

Fig. S10: The mean relative changes to stomatal conductance (*g*) from increase to pore area (*A*) and increases in pore depth (*d*) for both A) isotropic and B) anisotropic guard cell wall simulations. Per mesh values are given in C) and D). The diffusive resistance of an individual stomatal pore (*g*) is calculated using the geometric pore-diffusion model formalized by Parlange & Waggoner (1970):

$$g = \frac{DA}{d + \frac{\pi}{2}\sqrt{\frac{A}{\pi}}}$$

Methods S1

Pipeline for automatically extracting features from meshes

An automated procedure was developed to extract cross-sections from OBJ meshes generated from segmented confocal TIFF stacks using **MorphoGraphX**. Each OBJ file contains two mesh components, corresponding to the left and right guard cells of a stomatal complex. The position of the measurements are shown in **Fig. S2**.

1. **Shared wall detection:** The shared wall between the two guard cells was identified by finding vertices that are common to both mesh components within the same OBJ file. These shared vertices were then grouped into upper and lower wall regions using clustering.
2. **Midpoint cross-section:** A cross-section was extracted at the midpoint between the centres of the upper and lower shared wall regions.
3. **Circular end fitting:** At each guard cell tip, a circle with an area equivalent to that of the corresponding cross-section was fitted against the inner cell wall.
4. **Central spline fitting:** A spline was fitted through the centres of the circular tips and the midpoint cross-section, defining a central axis through the stomatal complex.
5. **Perpendicular sectioning:** Cross-sections were then extracted at regular intervals along the central spline, perpendicular to this central axis.

Methods S2

Pipeline for creating the idealised meshes

Idealised guard cell pair geometries were generated from baseline (0 MPa) empirical measurements using a custom Python routine. For each stomatal mesh ID, the initial cross-sectional minor radii were set to half the measured midsection major and minor lengths. Toroidal path radii were then derived to preserve the measured overall width and length. Two half-meshes were constructed using **Trimesh**, and the central pore area was estimated by rasterizing the XY projection at 0.01 μm resolution and flood-filling exterior pixels. The minor radii were iteratively adjusted, using a proportional gain of 0.1× the normalized pore area error, while recomputing path radii to maintain external dimensions. This process continued until the simulated pore area matched the empirical value within 0.5 μm^2, or a maximum of 10 iterations was reached. Converged meshes were exported as OBJ files for downstream analysis, while non-convergent cases were logged. For the circular variant, the major length was used for both minor radii to impose isotropy.

Video S1. Opening dynamics for a representative stomatal (onion) confocal image, comparing FEM model results from a mesh generated from the experimental guard cell geometry with meshes

generated from idealised guard cells with oval or circular starting cross-sections. Simulations show guard cell deformation and pore area change as turgor pressure is increased from 0 to 2 MPa.

Parlange, J.-Y., & Waggoner, P. E. (1970). Stomatal dimensions and resistance to diffusion. *Plant Physiology*, *46*(2), 337–342.
